# Coincidental occurrence of bilateral neonatal testicular torsion, with an extravaginal and a contralateral intravaginal testicular torsion

**DOI:** 10.1002/iju5.12649

**Published:** 2023-09-28

**Authors:** Yusuke Kirihana, Yuichi Sato, Junya Hata, Hitomi Imai, Yuki Yoshida, Kanako Matsuoka, Seiji Hoshi, Tomoyuki Koguchi, Soichiro Ogawa, Yoshiyuki Kojima

**Affiliations:** ^1^ Department of Urology Fukushima Medical University School of Medicine Fukushima Japan

**Keywords:** bilateral spermatic cord torsion, color Doppler ultrasonography, fasciotomy, intraoperative biopsy, neonatal spermatic cord torsion

## Abstract

**Introduction:**

We report a case of bilateral neonatal testicular torsion, with an extravaginal and a contralateral intravaginal testicular torsion.

**Case presentation:**

A 5‐day‐old boy with bilateral scrotal swelling and palpable induration was diagnosed with bilateral neonatal testicular torsion by color Doppler ultrasonography. The right testis was black with 360‐degree extravaginal torsion of the spermatic cord, and the left testis was brown with 90‐degree intravaginal torsion. We repaired the torsion and incised the tunica albuginea to reduce intratesticular pressure. The left testis became pink in color, but the right testis remained unchanged. Based on the pathological findings of the intraoperative biopsy of tissue specimens from both testes, we performed a right orchiectomy and preserved the left testis.

**Conclusions:**

Our experience suggests that testicular color improvement after fasciotomy and pathological findings of intraoperative testicular biopsy may indicate testicular preservation.

**Keynote message**

Rare case of bilateral neonatal testicular torsion, with an extravaginal and a contralateral intravaginal testicular torsion.

## Introduction

Testicular torsion in neonates differs from that in non‐neonates in terms of pathogenesis and clinical manifestations and has a very low testicular salvage rate.^1^, ^2^ About 90% of neonatal testicular torsion is extravaginal, which occurs before tunica vaginalis fixates to the scrotal subcutaneous tissue and has a different pathogenesis from intravaginal testicular torsion.^1^ Therefore, an extravaginal and a contralateral intravaginal testicular torsion rarely occur at the same time. We report a case of neonatal bilateral testicular torsion with simultaneous occurrences of an extravaginal torsion on one side and a contralateral intravaginal torsion on the other, beginning at 2 days of age, that allowed salvage of the left testis.

## Case presentation

**Fig. 1:**
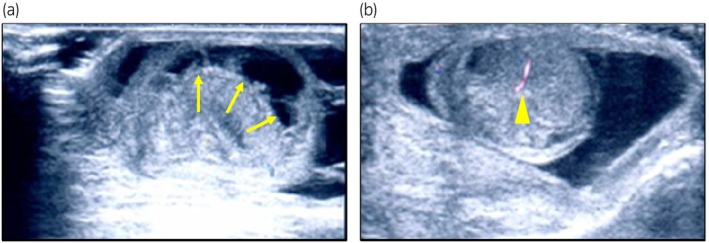
(a) Color Doppler ultrasonography showed fluid components and septal structures around the right testis with no blood flow (arrows). (b) Color Doppler ultrasonography showed fluid components and mosaic changes, which were considered to demonstrate decreased blood flow (arrowhead).

**Fig. 2:**
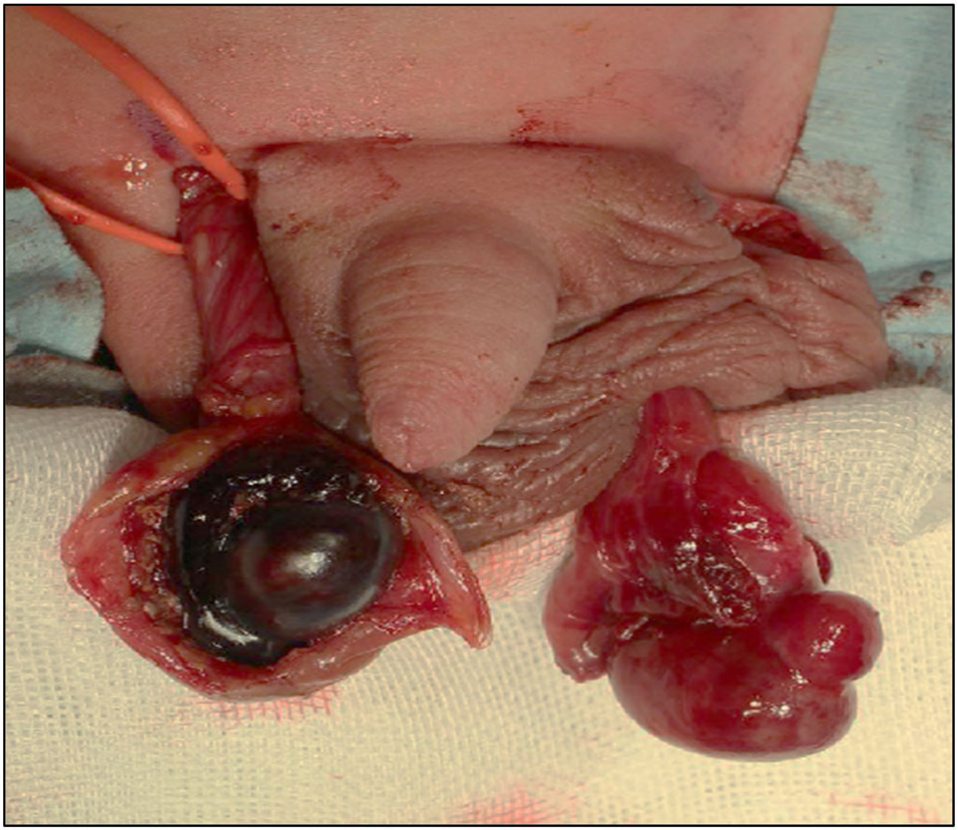
Intraoperative findings of both testes. The right testis was black with 360‐degree extravaginal torsion of the spermatic cord, and the left testis was brown with 90‐degree intravaginal torsion of the spermatic cord.

**Fig. 3:**
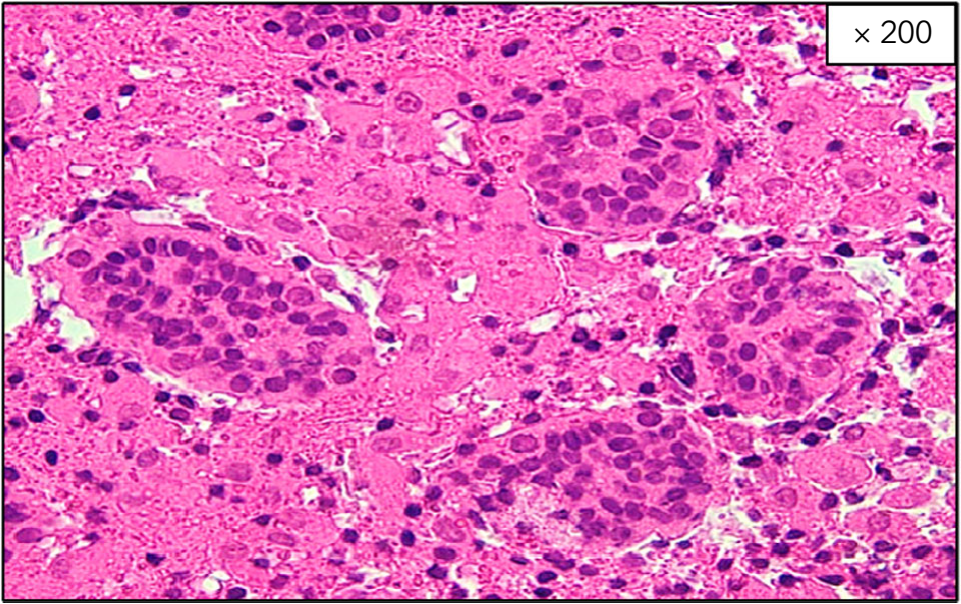
Histological findings of the right testis. Hematoxylin and eosin staining showed extensive infarction and necrosis.

**Fig. 4:**
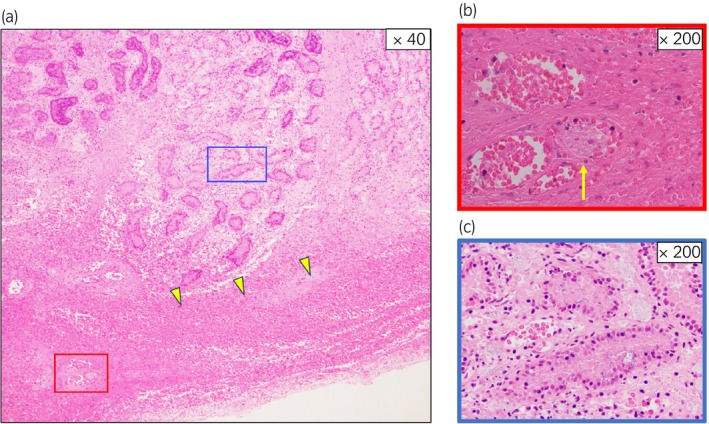
Histological findings of the left testis (a). Inflammation including hemorrhage (arrowheads), necrosis, and microthrombus (b: arrow) were found in the interstitial space, although the left testis showed some normal tubular structures (c).

The patient was born naturally at 39–5/7 weeks' gestation to a mother who received regular prenatal care. The pregnancy was uncomplicated, and the mother had no past medical history. The patient's Apgar scores were 9 at 1 min and 10 at 5 min after birth. The initial newborn physical examination showed no abnormalities. The genitalia were noted on the medical record as being normal. However, on Day 2 after birth, bilateral scrotal swelling and palpable induration of the testis were observed, and he was referred to our hospital on Day 5. The entire scrotum was swollen, the right testis was enlarged and palpable with induration, and the left testis was mildly enlarged and partially palpable with induration. Laboratory examinations were unremarkable. Color Doppler ultrasonography showed fluid components and septal structures around the right testis with no blood flow inside the testis (Fig. 1a). The left testis showed fluid components and mosaic changes, which were considered to demonstrate decreased blood flow (Fig. 1b). A bilateral neonatal testicular torsion was diagnosed, and surgical detorsion of the spermatic cord was performed. The right testis was black with a 360‐degree extravaginal torsion of the spermatic cord. On the other hand, the left testis was brown without extravaginal torsion. However, upon incising the tunica vaginalis, in addition to the accumulation of liquid components, a 90‐degree internal rotation was observed on the head side of the epididymis (Fig. 2). After repairing the torsion, although the right testis did not change color, suggesting no improvement, the left testis changed from black to pink. We incised the tunica albuginea of both testes to reduce intratesticular pressure, but the right testis did not improve in color after the fasciotomy. We performed a biopsy of both testes for tissue evaluation during the operation. Intraoperative pathology revealed extensive hemorrhagic necrosis of the right testis (Fig. 3). On the other hand, some seminiferous tubules of the left testis showed no significant damage, although inflammation with hemorrhage, necrosis, and microthrombus were found in the interstitial space (Fig. 4a–c). Therefore, we decided to perform a right orchiectomy and preserve the left testis. We closed the left testicular tunica albuginea using continuous sutures and securely positioned the left testicle within the scrotum. Two years after the surgery, the left testis was not atrophic, and color Doppler ultrasonography showed blood flow inside the testis.

## Discussion

Neonatal testicular torsion is a very rare disease.^1^, ^2^ It is classified into two major categories according to the time of onset: prenatal torsion, which occurs before birth, and postnatal torsion, which develops within the first 30 days after birth. The causes of neonatal testicular torsion are different from those of postnatal torsion. Most cases of neonatal testicular torsion are caused by twisting of the scrotal contents together with the tunica vaginalis before fixation to the scrotal subcutaneous tissue. Therefore, most cases are considered to be extravaginal, and previous reports have suggested that extravaginal testicular torsion accounts for 92% of all case, with the remaining 8% being intravaginal.^3^ Bilateral testicular torsions are extremely rare.^4^ To the best of our knowledge, there have been no reported cases of simultaneous occurrences of an extravaginal torsion on one side and a contralateral intravaginal torsion on the other.

In the present case, the types of testicular torsion suggested that the right testis had a failure of fixation to the scrotal subcutaneous tissue, and the left testis had an abnormal attachment to the tunica vaginalis.^5^ Pathological findings showed that the right testis had extensive infarction and necrosis, which was caused by the onset of extravaginal testicular torsion in the late fetal period. In addition, inflammation by right extravaginal testicular torsion may have spread over to the left testis, and the accumulation of fluid by the inflammation may have induced the consequent enlargement of the scrotal cavity and increased mobility of the left testis with abnormal attachment. This, in turn, caused intravaginal testicular torsion by external pressure during parturition.^6^

The rate of testicular sparing surgery for neonatal testicular torsion is very low, at 3–9%, compared to those for such torsion in infants or older children.^1^, ^2^, ^7^ Few cases of testicular sparing surgery for neonatal bilateral testicular torsion have been reported. In the present case, although the left testicular torsion was mild and improved in color after the fasciotomy, the pathological findings showed areas of hemorrhage, necrosis, and microthrombus. These results suggest that the left testis was ischemic due to torsion and then recovered spontaneously, which may have led to the sparing of the left testis.

## Conclusions

A coincidental occurrence of bilateral testicular torsion with different pathogenesis is very rare. In the present case, the spread of hemorrhagic necrosis suggested that the right testicular torsion occurred prenatally and the left testicular torsion occurred during parturition. In the case of a bilateral testicular torsion, it is difficult to decide whether to remove or preserve the testes. Our experience suggests that testicular color improvement after fasciotomy and intraoperative pathological findings of the remaining seminiferous tubules could be indicators of testicular preservation.

## Author contributions

Yusuke Kirihana: Conceptualization; writing – original draft. Yuichi Sato: Writing – review and editing. Junya Hata: Writing – review and editing. Hitomi Imai: Writing – review and editing. Yuki Yoshida: Writing – review and editing. Kanako Matsuoka: Writing – review and editing. Seiji Hoshi: Writing – review and editing. Tomoyuki Koguchi: Writing – review and editing. Soichiro Ogawa: Writing – review and editing. Yoshiyuki Kojima: Writing – review and editing.

## Conflict of interest

The authors declare no conflict of interest.

## Approval of the research protocol by an Institutional Reviewer Board

Not applicable.

## Informed consent

Not applicable.

## Registry and the Registration No. of the study/trial

Not applicable.
